# Multi‐hypothesis comparison of Farquhar and Collatz photosynthesis models reveals the unexpected influence of empirical assumptions at leaf and global scales

**DOI:** 10.1111/gcb.15366

**Published:** 2020-10-31

**Authors:** Anthony P. Walker, Abbey L. Johnson, Alistair Rogers, Jeremiah Anderson, Robert A. Bridges, Rosie A. Fisher, Dan Lu, Daniel M. Ricciuto, Shawn P. Serbin, Ming Ye

**Affiliations:** ^1^ Environmental Sciences Division and Climate Change Science Institute Oak Ridge National Laboratory Oak Ridge TN USA; ^2^ Environmental & Climates Sciences Department Brookhaven National Laboratory Upton NY USA; ^3^ Cyber & Applied Data Analytics Division Oak Ridge National Laboratory Oak Ridge TN USA; ^4^ National Center for Atmospheric Research Boulder CO USA; ^5^ Centre Européen de Recherche et de Formation Avancée en Calcul Scientifique (CERFACS) Toulouse France; ^6^ Computational Sciences & Engineering Division Oak Ridge National Laboratory Oak Ridge TN USA; ^7^ Department of Earth, Ocean, and Atmospheric Science Florida State University Tallahassee FL USA

**Keywords:** carbon assimilation, high‐resolution *A*–*C*_i_ curve, multi‐hypothesis modelling, photosynthesis, process sensitivity analysis, terrestrial biosphere model

## Abstract

Mechanistic photosynthesis models are at the heart of terrestrial biosphere models (TBMs) simulating the daily, monthly, annual and decadal rhythms of carbon assimilation (*A*). These models are founded on robust mathematical hypotheses that describe how *A* responds to changes in light and atmospheric CO_2_ concentration. Two predominant photosynthesis models are in common usage: Farquhar (FvCB) and Collatz (CBGB). However, a detailed quantitative comparison of these two models has never been undertaken. In this study, we unify the FvCB and CBGB models to a common parameter set and use novel multi‐hypothesis methods (that account for both hypothesis and parameter variability) for process‐level sensitivity analysis. These models represent three key biological processes: carboxylation, electron transport, triose phosphate use (TPU) and an additional model process: limiting‐rate selection. Each of the four processes comprises 1–3 alternative hypotheses giving 12 possible individual models with a total of 14 parameters. To broaden inference, TBM simulations were run and novel, high‐resolution photosynthesis measurements were made. We show that parameters associated with carboxylation are the most influential *parameters* but also reveal the surprising and marked dominance of the limiting‐rate selection *process* (accounting for 57% of the variation in *A* vs. 22% for carboxylation). The limiting‐rate selection assumption proposed by CBGB smooths the transition between limiting rates and always reduces *A* below the minimum of all potentially limiting rates, by up to 25%, effectively imposing a fourth limitation on *A*. Evaluation of the CBGB smoothing function in three TBMs demonstrated a reduction in global *A* by 4%–10%, equivalent to 50%–160% of current annual fossil fuel emissions. This analysis reveals a surprising and previously unquantified influence of a process that has been integral to many TBMs for decades, highlighting the value of multi‐hypothesis methods.

## Graphical abstract

Leaf photosynthesis models are the beating heart of global carbon cycle models, simulating the rhythms of CO_2_ transfer from the atmosphere to plants. Two key photosynthesis model variants are commonly used: the Farquhar and Collatz models, which vary in the way that some subprocesses are represented. Despite their almost ubiquitous use, the consequences of these alternative choices has never been formally investigated. Here we apply novel multi‐hypothesis modelling methods to investigate the influence of 14 parameters and four processes with alternative representations in photosynthesis models, finding the surprising dominance of a process that has not been extensively evaluated with data.

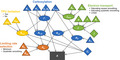

## 1 INTRODUCTION

As the gateway for carbon entry into terrestrial ecosystems, photosynthesis plays the keystone role in the biosphere of transferring atmospheric CO_2_ into terrestrial ecosystems. Since its inception 40 years ago the Farquhar et al. (1980; hereafter FvCB) model of C3 photosynthesis has revolutionized photosynthesis research (>5,000 citations, at time of writing). The FvCB model describes photosynthetic carbon assimilation (*A*) using a set of mathematically described hypotheses that represent the enzymatic subprocesses of photosynthesis and their integration, including: light‐stimulated electron transport, CO_2_ fixation in the Calvin–Benson cycle and photorespiration. The FvCB model is an integrated set of mathematically described hypotheses, a system hypothesis, that yields quantitative predictions to accurately describe the dynamics of *A* in response to incident radiation (*I*), carbon dioxide concentration (*C*_a_) and temperature. Observation, experiment and model‐based photosynthesis research has seen substantive advances due to the availability of this mathematically rigorous hypothesis. However, variants of the FvCB model exist, chief among them is that proposed by Collatz et al. (1991; hereafter CBGB). Differing hypotheses for three key subprocesses distinguish the models: (a) electron transport, (b) limiting process selection, and (c) triose phosphate use (TPU).

Terrestrial biosphere models (TBMs)—which simulate global land ecosystems and their role in the Earth System—rely on the FvCB and CBGB models (as well as various hybrids and additions to these core models) to simulate leaf‐scale photosynthesis and its response to global change, in particular, increasing *C*_a_. The CBGB model and hybrids with the FvCB model are employed by several prominent TBMs (Table 1; e.g. IBIS, JULES, CLM and ELM; Clark et al., 2011; Foley et al., 1996; Oleson et al., 2013). Yet despite the keystone role of photosynthesis in the biosphere and the wide variation in TBM simulations of photosynthesis (Anav et al., 2015; Rogers, Medlyn, et al., 2017), a rigorous, quantitative comparison of the FvCB and CBGB models has not been undertaken. In part this is because rigorous methods to compare and evaluate competing sets of hypotheses have not been available until recently.

**TABLE 1:** Factorial list of possible models from the given hypotheses

Model	Carboxylation	TPU	Limiting‐rate selection	Electron transport	TBMs, etc.
M1111	1. Equations (1), (4) and (5)	1. Absent	1. Equation (2)	1. Equation (6a)	FvCB,^a^ ORCHIDEE
M1211	1	2. Equation (7)	1	1	
M1121	1	1	2. Equations (3a,b)	1	
M1221	1	2	2	1	CLM4.5,^b^ FATES,^b^ ELM^b^
M1112	1	1	1	2. Equation (6b)	SDGVM; BETHY
M1212	1	2	1	2	
M1122	1	1	2	2	
M1222	1	2	2	2	
M1113	1	1	1	3. Equation (6c)	
M1213	1	2	1	3	CLM4.0,^b^ LM3^b^
M1123	1	1	2	3	
M1223	1	2	2	3	CBGB,^b^ CTEM,^b^ IBIS,^c^ JULES^b^

Abbreviations: TBMs, terrestrial biosphere models; TPU, triose phosphate use.

^a^ Strictly speaking FvCB used a different electron transport model but it was quickly superseded by Equation (6a) in 1984 and the original is not widely used.

^b^ While all of these models assume TPU limitation, they all assume *α*_tpu_ = 0 so that TPU limited *A* = 3TPU.

^c^ IBIS uses a unique TPU formulation (Foley et al., 1996).

Sensitivity analysis (SA) is used to determine the sensitivity of model output to variability in individual model components. Variability in model output can be introduced through a number of sources (Beven, 2016), two key sources are parameter choice and differences in mathematical representations of the multiple processes that a model simulates, e.g. photosynthetic electron transport. SA methods to assess model sensitivity to various *parameters* in TMBs are common (e.g. Dietze et al., 2014; Gupta & Razavi, 2018; Koven et al., 2019; Ricciuto et al., 2018; Zaehle et al., 2005) while SA methods to assess model output sensitivity to alternative *process representations* are rare. Parameter SA methods can be applied in the context of multiple models and sensitivity indexes averaged to get an overall assessment of parameter influence under model uncertainty (Dai & Ye, 2015). However, these methods miss a key element of model output variability—the difference in the means among models, or between‐model variation. Parameter SA can only account for within‐model variation, necessitating a process SA that is designed to account for both within (parameter) *and* between (process representation) model variation (e.g. Dai et al., 2017).

A further obstacle to rigorous process SA is that the majority of commonly used modelling codes are not sufficiently flexible to switch between all of the various hypotheses for all of the various processes under investigation. The Multi‐Assumption Architecture and Testbed (MAAT) has been designed to overcome this issue (Walker et al., 2018). MAAT is a hyper‐modular, multi‐hypothesis modelling framework designed to easily pose multiple alternative models by combining alternative mathematically described hypotheses for the processes that form the building blocks of a model, or system hypothesis (Walker et al., 2018). Through hyper‐modularity MAAT allows a factorial combination of each hypothesis across all processes and subprocesses, exploring the full range of possible models and ensuring that each representation of each process is evaluated against all other representations for all other processes, i.e. it is fully comprehensive. MAAT's ability to combine models at the scale of individual process hypotheses enables the application of rigorous process (SA) algorithms, such as that of Dai et al. (2017) which was designed to work with modelling codes like MAAT.

In this study we use MAAT to formally compare the leaf‐scale enzyme‐kinetic models of C3 photosynthesis by comparing the FvCB and CBGB model formulations. The choice of electron transport model, limiting‐rate selection, TPU limitation and parametric variability are examined. We ask the questions: (a) which processes are most influential for simulating carbon assimilation at various levels of atmospheric CO_2_ concentration and incident radiation, (b) which parameters are most influential, and (c) are the influential process and parameters different when considering absolute assimilation or the response of assimilation to a change in CO_2_? We further evaluate the outcome of this SA using global TBM simulations and measurements of leaf‐scale photosynthesis.

### 1.1 Comparison of the FvCB and CBGB models of photosynthesis

Enzyme‐kinetic models of photosynthesis (Farquhar et al., 1980) simulate net CO_2_ assimilation (*A—*µmol CO_2_ m^−2^ s^−1^) in response to CO_2_ concentrations in the intercellular airspace of the leaf (*C*_i_—Pa) and incident photosynthetically active radiation (*I*—µmol photons m^−2^ s^−1^). The model scales the gross carbon assimilation rate (*A*_g_—µmol CO_2_ m^−2^ s^−1^) to account for photorespiration, minus ‘dark’ respiration (*R*_d_—µmol CO_2_ m^−2^ s^−1^):

(1) $$A=A_{g}\left(1‐\Gamma_{*}/C_{i}\right)‐R_{d},$$

where $\Gamma_{*}$ is the photorespiratory CO_2_ compensation point (Pa), the *C*_i_ at which *A*_g_ is equal to the rate of CO_2_ release from oxygenation. *A*_g_ is simulated as a change point model (Gu et al., 2010) where one of two (FvCB) or three (CBGB) potentially limiting processes (*A*_c,g_, *A*_j,g_, and *A*_p,g_—µmol CO_2_ m^−2^ s^−1^), described in detail below, are selected. FvCB simply identifies the minimum of the potentially limiting rates:

(2) $$A_{g}=\min\left\{A_{\text{c,g}},A_{\text{j,g}},A_{\text{p,g}}\right\},$$

resulting in discontinuities in the derivative of the *A–C*_i_ or *A–I* curves at the change points where *A*_c,g_ = *A*_j,g_ and *A*_j,g_ = *A*_p,g_. In order to ‘*introduce a more realistic, gradual transition from one limitation to another, and to allow for some co‐limitation*’, CBGB proposed non‐rectangular hyperbolic (quadratic) smoothing between the three potentially limiting rates:

(3a) $$0=\theta_{\text{cj}}A_{\text{cj,g}}^{2}‐\left(A_{\text{c,g}}+A_{\text{j,g}}\right)A_{\text{cj,g}}+A_{\text{c,g}}A_{\text{j,g}},$$

(3b) $$0=\theta_{\text{cjp}}A_{g}^{2}‐\left(A_{\text{cj,g}}+A_{\text{p,g}}\right)A_{g}+A_{\text{cj,g}}A_{\text{p,g}},$$

where *A*_cj,g_ is a latent variable resulting from smoothing between *A*_c,g_ and *A*_j,g_. Parameters *θ*_cj_ and *θ*_cjp_ (*θ* and *β* in CBGB's original notation) are curvature parameters that take a value 0–1 with lower values leading to greater smoothing. The FvCB method is a special case of the CBGB method where both *θ*_cj_ and *θ*_cjp_ take the value 1, while if *θ*_cj_ and *θ*_cjp_ take the value 0 smoothing becomes rectangular hyperbolic (Johnson & Thornley, 1984).

*A*_c,g_, *A*_j,g_ and *A*_j,g_ are modelled as Michaelis–Menten functions of *C*_i_. For *A*_c,g_, *V*_cmax_ (µmol CO_2_ m^−2^ s^−1^) determines the asymptote:

(4) $$A_{\text{c,g}}=\frac{V_{\text{cmax}}C_{i}}{C_{i}+K_{c}\left(1+O_{i}/K_{o}\right)},$$

where *O*_i_ is the internal O_2_ partial pressure (kPa), and *K*_c_ (Pa) and *K*_o_ (kPa) are the Michaelis–Menten half‐saturation constants of the RuBisCO enzyme for CO_2_ and for O_2_. For *A*_j,g_, the asymptote is proportional to the electron transport rate (*J—*µmol e m^−2^ s^−1^) where:

(5) $$A_{\text{j,g}}=\frac{J}{4}\frac{C_{i}}{\left(C_{i}+2\Gamma_{*}\right)},$$

A number of hypotheses to represent *J* exist, most commonly used are the following three. Based on Smith (1937), two representations of *J* saturate at a maximum electron transport rate (*J*_max_), (a) Farquhar and Wong (1984) used non‐rectangular smoothing (Equation 6a), (b) Harley et al. (1992) use an alternative non‐rectangular hyperbola (Equation 6b), while (c) CBGB proposed a linear model that has no maximum (Equation 6c) respectively:

(6a) $$0=\theta_{j}J^{2}+a\alpha_{i}IJ_{\max}J+a\alpha_{i}IJ_{\max},$$

(6b) $$J=\frac{a\alpha_{i}I}{{\left[1+{\left(\frac{a\alpha_{i}I}{J_{\max}}\right)}^{2}\right]}^{0.5}},$$

(6c) $$J=a\alpha_{i}I,$$

where *a* is the leaf absorptance (the fraction of *I* absorbed by the leaf, unitless), *α*_i_ is the intrinsic quantum efficiency of electron transport (the number of electrons transported per absorbed photon, e photon^−1^) and *θ*_j_ is a non‐rectangular hyperbola smoothing parameter. *aα*_i_ is the apparent quantum efficiency of electron transport (electrons transported per incident photon). Following FvCB, in this study we define *α*_i_ as 0.5(1 − *f*), where *f* is the fraction of photons absorbed by the leaf but not absorbed by the light harvesting complexes, and 0.5 represents the requirement of two photons for full linear transport of a single electron.

Subsequent to the development of the FvCB model, a third potential limitation was identified under high *C*_a_ and high irradiance (*I*). TPU in sucrose and starch synthesis releases phosphate needed for the regeneration of RuBP, thus low rates of sucrose and starch synthesis can limit RuBP regeneration and therefore *A* (Sharkey, 1985). CBGB proposed this additional rate‐limiting cycle in their model (*A*_p,g_), which was refined (von Caemmerer, 2000) to account for reversed sensitivities of *A* to *C*_i_ and *O_i_* in the TPU limiting state (Harley & Sharkey, 1991):

(7) $$A_{\text{p,g}}=\frac{3{\text{TPU C}}_{i}}{C_{i}+\left(1+3\alpha_{\text{tpu}}\right)\Gamma_{*}},$$

where *α*_T_ is the fraction of glycolate exported but not returned to the chloroplast during photorespiration. CBGB assumed a closed photorespiratory cycle (*α*_T_ = 0) such that multiplication by the first term in Equation (1) yields 3TPU.

## 2 METHODS

As described above, the MAAT (Walker et al., 2018; https://github.com/walkeranthonyp/MAAT) is a hyper‐modular, multi‐hypothesis modelling framework. MAAT is written in R (R Core Team, 2020) and provides a general framework and code structure for building models that allows for new processes to be added easily. Higher‐level ‘system models’ integrate multiple processes into a coherent representation of a given system. A number of these system models have been coded into MAAT and in this study we use the leaf‐scale enzyme‐kinetic model of photosynthesis (Walker et al., 2018). MAAT also encodes process and parameter SA algorithms. In this study we used MAAT (tag: v1.2.1_walkeretal2020_GCB, commit hash: 09b1479).

### 2.1 Additional model details

**FIGURE 1:**
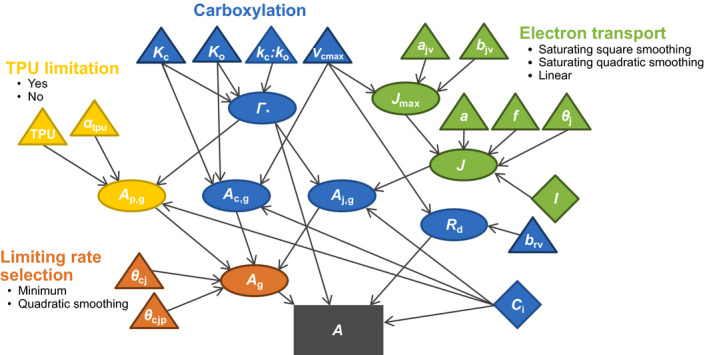
Diagram of photosynthesis model to calculate net carbon assimilation (*A*, μmol m^−2^ s^−1^, grey rectangle). Inputs (diamonds), parameters (triangles), functions (ellipses) and target state variable (rectangle) are shown, as is the breakdown on the model into the four processes (colours; limiting‐rate selection, carboxylation, electron transport and triose phosphate use). Arrows represent the flow of information from distal parts of the model (inputs and parameters), through intermediate functions to the end goal. Concept modified slightly from Coon et al. (2016)

Figure 1 shows a dependency diagram of the general C3 photosynthesis model. Parameters, state parameters (variables that are calculated during model execution but are not the main model state), state variables (i.e. carbon assimilation rate) and their dependencies are shown categorized by the four processes: limiting‐rate selection, electron transport, TPU and carboxylation. Each process is composed of multiple parameters and, excepting carboxylation, multiple ways in which they can be represented. Some processes have more than one mathematical function in their representation, e.g. carboxylation which includes Equations (1), (4), (5) and (8).

Note that in the original studies, FvCB and CBGB describe the enzyme‐kinetic models using different units and slightly different parameter definitions. In this study we use the unification of definitions and units presented in Walker et al. (2018) that are built upon Gu et al. (2010) and predominantly follow FvCB. Building on the model details presented in the introduction, $\Gamma_{*}$ can be simulated as a function of *K*_c_ and *K*_o_:

(8) $$\Gamma_{*}=\frac{k_{o}K_{c}O_{i}}{2k_{c}K_{o}},$$

where *k*_o_ and *k*_c_ are the turnover numbers for the oxygenase and carboxylase functions of RuBisCO. At 25°C *k*_o_ is 0.21 times *k*_c_, and their activation energies are the same so this ratio is preserved across a range of temperatures (Farquhar et al., 1980).

*J*_max_ can be represented in a number of ways but many studies have demonstrated the tight correlation between *J*_max_ and *V*_cmax_ (Leuning et al., 1995; Walker, Beckerman, et al., 2014; Wullschleger, 1993) and for the basis of this study we use the commonly employed linear relationship based on the relationship proposed by (Wullschleger, 1993):

(9) $$J_{\max,25}=a_{\text{jv}}+b_{\text{jv}}V_{\text{cmax},25},$$

where *a*_jv_ and *b*_jv_ are calculated from linear regression of *J*_max,25_ on *V*_cmax,25_ (which are *J*_max_ and *V*_cmax_ at a reference temperature of 25°C). We use a similar relationship to calculate TPU:

(10) $${\text{TPU}}_{25}=b_{\text{tv}}V_{\text{cmax},25}.$$

We focus on the core components of the FvCB and CBGB models and do not consider choices of CO_2_‐diffusion resistance models (i.e. leaf boundary layer, stomata and internal; see Collatz et al., 1991; Walker et al., 2018) nor temperature response models, which would substantially increase scope. Further, while stomatal models do influence *C*_i_, the response to *I* and *C*_a_ of many stomatal models used by TBMs is similar, i.e. *g*_s_ *= f*(*A/C*_a_). In order to preserve realism in the calculation of *A* we used the unified stomatal model (Medlyn et al., 2011) with a *g*_1_ of 4.3, the global C3 mean (Lin et al., 2015), and a typical value of 0.01 mol m^−2^ s^−1^ for *g*_0_. We assume that leaf internal resistance and leaf boundary layer resistance are 0, vapour pressure deficit is 1 kPa, and no soil water limitation. Leaf temperature was set to a standard of 25°C (which assumes that all temperature sensitive parameters were at 25°C reference values).

To generate *C*_a_ and *I* response curves, the two models were run from a *C*_a_ of 50–1,500 µmol/mol in 50 µmol/mol increments at *I* of 960 μmol m^−2^ s^−1^, and from *I* of 10–1,960 μmol m^−2^ s^−1^ in increments of 50 μmol m^−2^ s^−1^ at *C*_a_ of 400 µmol/mol.

### 2.2 Sensitivity analysis

We use statistical, variance‐based process SA and parameter SA which both rely on an ensemble of model simulations to calculate model output variance and ascribe this variance to variation in processes and parameters. The photosynthesis model process SA was broken into four processes: limiting‐rate selection (two representations, Equations (2), (3a), (3b) or (2), (3a), (3b)), electron transport (three representations, Equations (6a), (6b), (6c)), TPU limitation (included or not included, Equation 7) and carboxylation (one representation, Equation 4). Factorial combination of the alternative process representations gives a total of 12 individual models. For both the process SA and parameter SA, 14 parameters were varied ±10% from a central, commonly used value (Table 2). Thus the model ensemble comprises 12 individual models and variation of 14 parameters.

**TABLE 2:** Comparison of the parameters used in the original papers by Farquhar and Collatz unified to common units

Parameter	Equations	Description	Units	FvCB	CBGB	SA range
*V*_cmax_	4, 9 and 10	Maximum RuBisCO carboxylation rate	μmol CO_2_ m^−2^ s^−1^	98	200	45–55
*K*_c_	4 and 8	Michaelis–Menten constant of RuBisCO for CO_2_	Pa	46	30	36.4–44.5
*K*_o_	4 and 8	Michaelis–Menten constant of RuBisCO for O_2_	kPa	33	30	25.1–30.6
*k*_o_:*k*_c_	8	Ratio of RuBisCO turnover numbers for O_2_ and CO_2_	—	0.21	0.38^a^	0.19–0.23
*J*_max_	6a, 6b and 9	Maximum electron transport rate	μmol e m^−2^ s^−1^	210	na	na
*a*_jv_	9	Intercept of *J*_max_ to *V*_cmax_ relationship	μmol e m^−2^ s^−1^	na	na	26.2–32.0
*b*_jv_	9	Slope of *J*_max_ to *V*_cmax_ relationship	e CO_2_^−1^	na	na	1.467–1.804
*a*	(6a), (6b), (6c)	Leaf absorbtance of visible solar radiation	—	na (0.80)^b^	0.86	0.72–0.88
*f* (*α*_i_ = 1 − *f*)	(6a), (6b), (6c)	Fraction of absorbed light not absorbed by photosystems	—	0.23	0.52^c^	0.207–0.253
*θ*_j_	6a	Electron transport smoothing	—	na (0.67)^b^	na	0.81–0.99
*θ*_cj_	3a	Assimilation rate smoothing 1	—	na	0.95	0.81–0.99
*θ*_cjp_	3b	Assimilation rate smoothing 2	—	na	0.98	0.81–0.99
TPU	7	Triose phosphate utilisation	μmol CO_2_ m^−2^ s^−1^	na	0.167 *V*_cmax_	0.15–0.183 *V*_cmax_
*α*_tpu_	7	Fraction of phosphate exported from chloroplast not returned	—	na	na	0.45–0.55
*R*_d_	1	Dark respiration	μmol CO_2_ m^−2^ s^−1^	1.1	0.015 *V*_cmax_	0.0135–0.0165 *V*_cmax_

Abbreviation: TPU, triose phosphate use.

^a^ Calculated from Collatz CO_2_:O_2_ specificity ratio, τ in their notation, of 2,600 where *k*_o_:*k*_c_ = *K*_o_/(*K*_c_τ).

^b^ Parameters were not originally specified in Farquhar but values in parentheses featured in Farquhar and Wong (1984).

^c^ Calculated from Collatz value of intrinsic quantum yield, α in their notation, of 0.08 where 0.5(1 − *f*)/4 = *α*.

Both methods calculate sensitivity indexes that represent the proportion of variance in model‐ensemble output (both between and within‐model variance for the process SA, and just within‐model variance for the parameter SA) caused by variance in either a specific process or a specific parameter. Process SA provides a quantitative assessment of the influence of processes on model output and includes both variation caused by alternative hypotheses for the mechanics of a given process, and variation caused by parameters within a given process, i.e. between‐model and within‐model variability (Dai et al., 2017). Parameter SA can assess the influence of individual parameters on within‐model variance. The parameter SA method does not account for variance caused by the different central tendencies (means) of each model combination. For process SA the algorithm of Dai et al. (2017) was used and for parameter SA the algorithm of Saltelli et al. (2010) was used (see Walker et al., 2018 for additional description of both methods).

In this analysis we calculate and focus on first order sensitivity indexes, analogous to main effects in ANOVA. We do not calculate two‐way or higher order interactions among processes or parameters. While some interactions are likely to be interesting, the sum of first order sensitivity indexes sum to around 0.95 in many cases indicating that over 95% of variance in model output was explained by first order effects.

For both analyses, we investigated sensitivities of simulated carbon assimilation (*A*, Equation 1) across a number of environmental scenarios that were a factorial combination of atmospheric CO_2_ (*C*_a_; 280, 400 and 600 µmol/mol) and incident light (*I*; 200, 500, and 1,000 µmol photons m^−2^ s^−1^) conditions. The sensitivity of the absolute assimilation response (Δ*A*) to changes in *C*_a_ (from preindustrial to present‐day, 280–400 µmol/mol, and from present‐day to projected future concentrations, 400–600 µmol/mol) were also calculated under the three incident light conditions. Variance‐weighted means of the sensitivity indexes across different environment combinations allow us to quantify the general influence of a process or parameters across environment combinations. For parameters this can also be done across model combinations, but again still only account for within model variance.

Parameter samples were drawn from uniform distributions and were multi‐dimensional (all parameters varied together) but no covariance among parameters was assumed. CBGB used values of 0.95 and 0.98 for *θ*_cj_ and *θ*_cjp_, see table A1 in CBGB. Note values are switched in the text of CBGB, i.e. 0.98 and 0.95 for *θ*_cj_ and *θ*_cjp_. Other values have since been used by TBMs; e.g. 0.9 in IBIS (Foley et al., 1996) and 0.83 in JULES (Clark et al., 2011). The possible values for these smoothing parameters are from 0 ≤ *θ ≤* 1 and so to maintain values within this range and preserve the ±10% variation in all parameters, we use a central value of 0.9 for *θ*_cj_ and *θ*_cjp_. These data are publically available (Walker, Lu, et al., 2020).

To assess convergence in sensitivity index calculations, preliminary SAs were run with an *n* of 1,000 for the process SA and 1,000,000 for the parameter SA. Subsampling and bootstrapping indicated that an *n* of 300 for the process SA and 300,000 for the parameter SA were ample to achieve convergence (standard deviations of the sensitivity indexes were less than 0.001). The results of the SA shown in this study were generated using these smaller values of *n*, resulting in a total of 4,320,000 executions for the process SA and 50,400,000 for the parameter SA. These total number of executions are larger than *n* as *n* is the base number of samples and the full SAs require multiple sets of iterations that are a function of the number of model combinations and parameters investigated (see Walker et al., 2018).

### 2.3 Estimation of *θ*_cj_ from high‐resolution *A*–*C*_i_ curves

High‐resolution *A–C*_i_ curves (Anderson et al., 2020) were used to evaluate limiting‐rate selection hypotheses. *Populus canadensis* Moench. [deltoides × nigra] clone OP367 was grown outside at Brookhaven National Laboratory, NY, USA in 200 L pots containing 52 Mix (Conrad Fafard, Inc.). Hardwood cuttings were planted on 1 May 2019 and plants were watered to field capacity two to three times a week. Photosynthetic CO_2_ response (*A–C*_i_) curves were measured using a LI‐6800 Portable Photosynthesis System (LI‐COR) in June 2019.

Preliminary measurements identified saturating *I* and *C*_a_ where photosynthesis transitioned from RuBP saturated (*A*_c,g_) to RuBP limited (*A*_j,g_) photosynthesis. These preliminary measurements informed a commonly used *A–C*_i_ response protocol (Rogers, Serbin, et al., 2017), developed to include a high density of measurements around the transition point (when *A*_c,g_ = *A*_j,g_). Leaves were first acclimated to chamber conditions (*I* = 2,000 μmol m^−2^ s^−1^, *C*_a_ = 400 μmol/mol, flow rate = 600 μmol/s, relative humidity = 70%–75%, leaf temperature = 30°C) and measurements began once steady‐state gas exchange was achieved. *C*_a_ was taken from 400 to 50 µmol/mol then returned to a conservative estimate of the start of the transition zone (305 µmol/mol) and raised progressively in 5 µmol/mol increments to 1,000 µmol/mol (a value comfortably higher than the end of the transition zone). *C*_a_ was then raised in larger increments to capture the full extent of a standard *A–C*_i_ curve.

Bayesian machine‐learning, Markov chain Monte Carlo (MCMC), algorithms were used to numerically approximate the posterior distribution of the smoothing parameter, *θ*_cj_ (Equation 3a), and *V*_cmax_ and *J*_max_, from these high‐resolution *A–C*_i_ curves. Numerical approximation is achieved by randomly sampling from a specified prior distribution, stochastically generating a proposal for the parameters to be estimated, evaluating the likelihood of the proposed parameter values against observed data and iterating the generation of new proposals to search the prior parameter space until convergence of the joint posterior distribution is reached. MCMC algorithms are then further iterated postconvergence to sample the joint posterior distribution. For this analysis, the Differential Evolution Adaptive Metropolis (DREAM) algorithm (Vrugt et al., 2009) was chosen due to its efficient search of parameter space and rapid convergence relative to other MCMC algorithms.

The DREAM algorithm optimizes parameter space sampling in several ways (Vrugt, 2016). First, the algorithm employs multiple parallel MCMC chains and parameter proposals are generated from randomly selected chain pairs. A scaling factor is used to scale the ‘jump’ distance of the new proposal from the previously accepted proposal. At approximately every fifth iteration the scaling factor is set to 1 to avoid convergence in local minima. The algorithm avoids the inefficiency that arises from updating all parameters of a chain simultaneously by updating only a randomly selected subset (the ‘crossover’) of the parameters on a chain with optimized probability. Outlier chains are identified based on the interquartile range of the posterior likelihood and replaced with the sample history of another randomly chosen non‐outlier chain.

Coded within the MAAT software framework, the DREAM algorithm used the high‐resolution *A–C*_i_ data to formally estimate the parameter values for *V*_cmax,25_, *J*_max,25_, and *θ*_cj_ for each leaf. Uniform priors were used, taking the values 100–200 μmol CO_2_ m^−2^ s^−1^ for *V*_cmax,25_, 70–400 μmol e m^−2^ s^−1^ for *J*_max,25_, and 0.9–1.0 for *θ*_cj_ (unitless). Environmental variables were set to the conditions used to generate the *A–C*_i_ curves (see above). Given the high rates of photosynthesis in these plants, temperature optima of *V*_cmax_ and *J*_max_ were assumed high at 35 and 30°C. Seven Markov chains were run for 80,000 iterations. The standard error probability density function with independent and identically distributed (iid) error residuals was used to compute the log‐likelihood of the proposal generation. On completion of the 80,000 iterations convergence was determined using the Gelman and Rubin (1992) R‐statistic. Preconvergence samples were discarded yielding 25,000 postconvergence samples on each chain, these were then thinned to 1% to remove auto‐correlation.

### 2.4 TBM simulations

We use three TBMs to test the impact of quadratic smoothing on global GPP simulations: (a) the Energy Exascale Earth System Model (E3SM) land model (ELM; release: v1.1.0; Burrows et al., 2020), a coupled carbon, nitrogen and phosphorus model with sun/shade big‐leaf canopy photosynthesis scaling. (b) The Functionally Assembled Terrestrial Ecosystem Simulator (FATES, tag: sci.1.30.0_api.8.0.0; Koven et al., 2019), coupled with the Community Land Model (CLM, version 5; Lawrence et al., 2019), a carbon‐only vegetation demography model with multi‐leaf and multi‐canopy layers for scaling photosynthesis, and with a leaf area index (LAI) optimization scheme. (c) The Sheffield Dynamic Global Vegetation Model (SDGVM, tag: Walkeretal2020_GCB), a carbon‐only model with multi‐leaf‐layer canopy photosynthesis scaling and also with an LAI optimisation scheme (Walker et al., 2017; Woodward & Lomas, 2004). The leaf photosynthesis model in both ELM and FATES is based on CBGB but with an electron transport function that includes a *J*_max_ term (Equation 3a). While SDGVM uses the FvCB model with a similar electron transport function (Equation 3b). For more information about the models, see Notes S1.

Models were run each using their common configurations and input datasets to allow for a cross‐section of results. The goal was to assess the possible impacts of smoothing in global TBM simulations across a range of model types rather than to quantify the exact impact under a specific set of conditions. Thus, commonly used configurations allowed a broader sampling of the possible model configuration space. The only strict protocol was to use consistent values for the smoothing parameters. Two simulations were conducted: a simulation using smoothing parameters (0.95 for *θ*_cj_ and 0.98 for *θ*_cjp_) and a no‐smoothing simulation in which the minimum of limiting rates were taken or where smoothing was effectively disabled by setting their parameter values to 1 or 0.9999 (for both). Decadal average annual GPP from the two simulations were then compared to determine the impact of the smoothing parameters. Model results were re‐gridded to a common 0.5° × 0.5° spatial grid, using bilinear interpolation where necessary (ELM). A 0.5° land mask was then applied to constrain model output to a common areal extent on which to base annual calculations and maps of global GPP (Walker, Fisher, et al., 2020).

## 3 RESULTS

**FIGURE 2:**
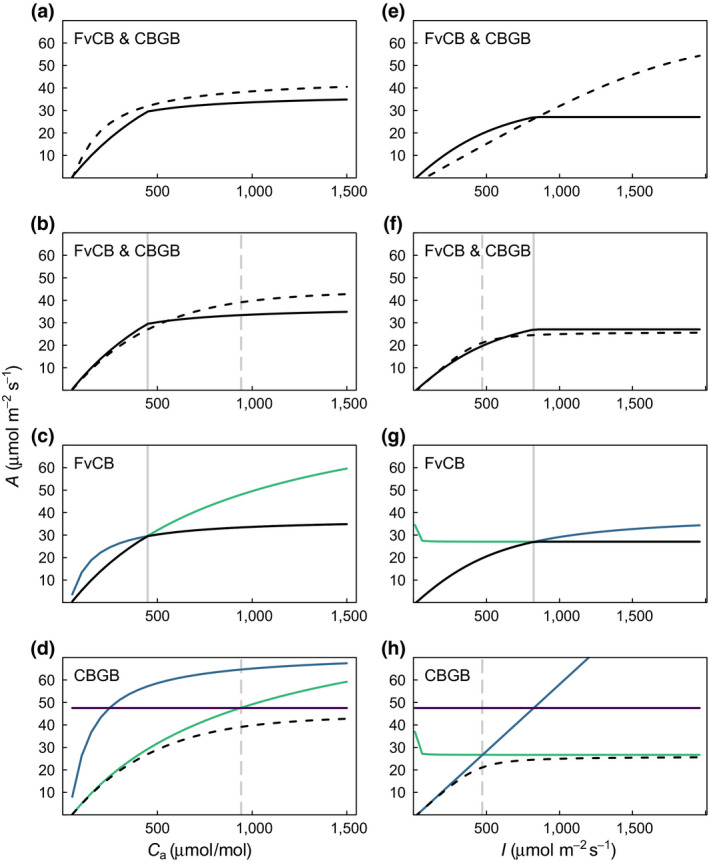
Comparison of FvCB (solid black line) and CBGB (dashed black line) calculated carbon assimilation (*A*, μmol m^−2^ s^−1^) in response to *C*_a_ (left column) and *I* (right column) for the models, (a) and (e) in their original state and parameterization (Table 1), (b) and (f) in their original state but with common parameterization (using FvCB parameters Table 1). (c and g) FvCB and (d and h) CBGB showing the two or three potentially limiting rates *A*_c,g_ (green), *A*_j,g_ (blue) and *A*_p,g_ (purple) in addition to *A* (black solid or dashed). Vertical grey lines show the transition points between limiting rates for the FvCB (solid) and CBGB (dashed) models. Common parameterizations are for shared parameters, i.e. the quadratic smoothing parameters are not common as quadratic smoothing is not considered by FvCB even though selection of the minimum can be represented by a special case of quadratic smoothing

In their original parameterizations, the *C*_a_ response of CBGB is smoother and more sensitive than FvCB at both low and high *C*_a_ (Figure 2a). With unified parameters the models are similar at low to intermediate *C*_a_ but at high *C*_a_ the CBGB model is again more sensitive to *C*_a_ and the difference in *A* approaches 10 μmol m^−2^ s^−1^ at 1,500 µmol/mol (Figure 2b). Comparison of *A* implied by each of the two or three potentially limiting rates (i.e. calculating *A* from *A*_c,g_, *A*_j,g_, and *A*_p,g_ in Equation 1) explains these responses (Figure 2c,d).

*A* in the FvCB model tracks exactly *A* implied by one of the two potentially limiting rates and consequently shows a sharp transition at the point where carboxylation‐limited and light‐limited rates are equal (Figure 2c). Above this transition point light limits *A*, though some sensitivity to *C*_a_ remains due to competitive‐inhibition of photorespiration. For the CBGB model, *A* also closely tracks the carboxylation‐limited rate at low *C*_a_. Between 300 and 400 µmol/mol *A* begins to deviate from any *A* implied by the three potentially limiting rates (Figure 2d); a consequence of quadratic smoothing (Equations 3a,b). The largest departure of *A* from any of the implied rates (a difference close to 10 μmol m^−2^ s^−1^) is at the transition point which, notably, is between the carboxylation‐limited rate and the TPU‐limited rate, not the light‐limited rate. The light‐limited rate is much greater than *A*, by over 20 μmol m^−2^ s^−1^ for most of the range in *C*_a_ concentrations (Figure 2d). Thus the continued sensitivity of *A* to *C*_a_ above the transition point results not from suppression of photorespiration, but from less influence of quadratic smoothing as TPU limitation becomes the dominant limiting rate (reduction of *A* by smoothing increases as limiting rates become more similar, discussed in more detail below).

With their original parameterizations the light responses of the two models are very different (Figure 2e). The FvCB model shows a curve similar in nature to its *C*_a_ response, a steep increase, an abrupt transition followed by saturation; while the CBGB model shows a close to linear increase across the range of *I*. The linear response to *I* of CBGB results from the (a) very high *V*_cmax_ (200 μmol m^−2^ s^−1^, Table 2) in the original parameterization, which prevents *C*_a_ limitation at 400 µmol/mol across the range of *I*, and (b) the absence of a *J*_max_ term in the electron transport response (Equation 6c) which therefore hypothesizes a linear response of electron transport to *I*. At common parameter values (Figure 2f), the curves are much more similar. This is because at the lower, common *V*_cmax_ (98 μmol m^−2^ s^−1^) *A*_c_ becomes limiting at 400 µmol/mol. For light, *A* in both models tracks *A* implied by the potentially limiting rates more closely than for CO_2_ (Figure 2g,h). This is because the transition between the implied rates is more abrupt and therefore the range of *I* where smoothing occurs is narrower. The greatest difference among the two models are in their light‐limited rates, FvCB shows strong non‐linearity and saturates (due to the *J*_max_ term in Equation 6a) while CBGB shows a linear response to light (Equation 6). Curvature in the light response of realised assimilation rates come from the *θ*_j_ parameter for the FvCB model and the *θ*_cj_ parameter for the CBGB model.

### 3.1 SA of assimilation (*A*)

**FIGURE 3:**
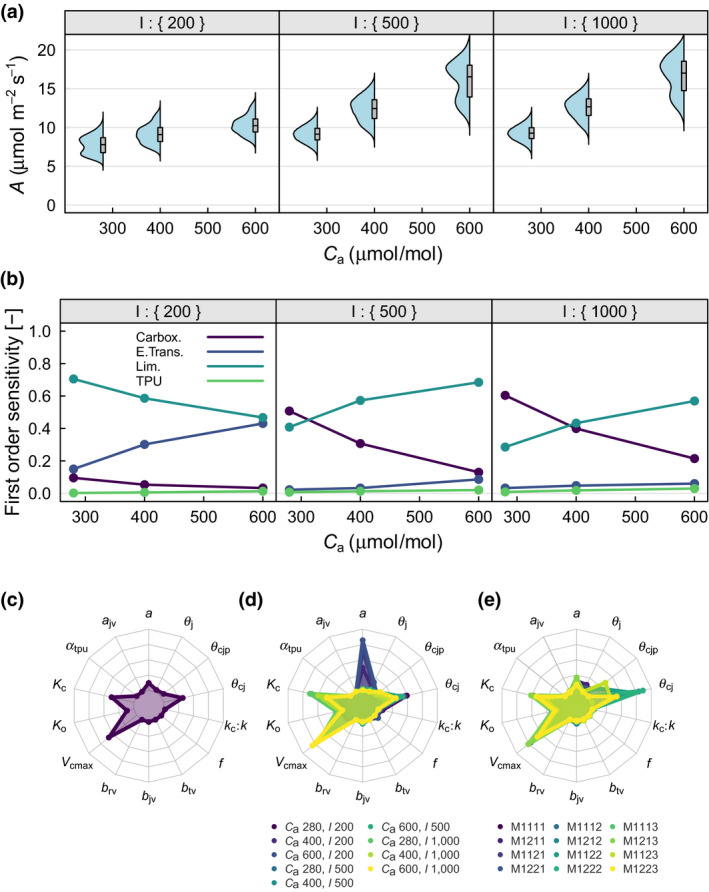
Sensitivity of carbon assimilation (*A*, μmol m^−2^ s^−1^) to variability in processes and parameters across various *C*_a_ and *I* environmental conditions. (a) Semi violin plots showing distributions of *A* against *C*_a_ (μmol/mol, *x*‐axis) and *I* (μmol m^−2^ s^−1^, panels) boxes represent the interquartile range and median, whiskers the full range. (b) First order sensitivity index of *A* to variability in the four processes against *C*_a_ (μmol/mol, *x*‐axis) and *I* (μmol m^−2^ s^−1^, panels). (c) First order sensitivity index of *A* to variability in the 14 parameters, indexes integrated across the 12 models and nine environmental conditions. (d) First order sensitivity index of *A* to variability in the 14 parameters, indexes integrated across the 12 models and colour coded for each of the nine environmental conditions. (e) First order sensitivity index of *A* to variability in the 14 parameters, indexes integrated across the nine environmental conditions and colour coded for each of the 12 models

Figure 3a shows distributions of *A* when varying representations of the three processes and the values of the 14 parameters (Table 3) across nine combinations of *C*_a_ and *I* (i.e. different environmental conditions). As *C*_a_ increases, A and variance of *A* increases (Figure 2a; Table 1). Distributions are primarily unimodal, though several bimodal distributions are apparent.

**TABLE 3:** First order sensitivity to processes

Variable	*C*_a_	*I*	Mean	Variance	Carbox.	E.Trans.	Lim.	TPU
*A*	int^a^	int	11.49	2.59	0.22	0.10	0.57	0.02
	280	200	7.77	1.46	0.10	0.15	0.71	0.00
	400	200	9.15	1.54	0.05	0.03	0.59	0.01
	600	200	10.31	1.49	0.03	0.43	0.47	0.01
	280	500	9.16	1.11	0.51	0.02	0.41	0.01
	400	500	12.38	2.61	0.31	0.03	0.57	0.01
	600	500	16.08	6.19	0.13	0.09	0.68	0.02
	280	1,000	9.28	0.99	0.60	0.03	0.29	0.01
	400	1,000	12.61	2.21	0.40	0.05	0.43	0.02
	600	1,000	16.68	5.76	0.21	0.06	0.57	0.03
∆*A*	int	int	2.81	0.50	0.05	0.13	0.65	0.03
	280–400^b^	200	1.38	0.10	0.10	0.50	0.07	0.01
	400–600	200	1.16	0.04	0.11	0.32	0.37	0.05
	280–400	500	3.22	0.40	0.07	0.05	0.76	0.02
	400–600	500	3.70	1.11	0.02	0.18	0.64	0.03
	280–400	1,000	3.32	0.32	0.11	0.07	0.64	0.04
	400–600	1,000	4.07	1.02	0.05	0.07	0.69	0.04

Abbreviation: TPU, triose phosphate use.

^a^ Integrated across environmental scenarios.

^b^ For a change in Ca from 280 to 400 µmol/mol.

Across all environmental conditions, limiting‐rate selection was responsible for 57% of the variation in *A*, carboxylation responsible for 22%, electron transport 10% and TPU 2% (Table 3). The strong influence of limiting‐rate selection was borne out across the majority of environmental conditions (Figure 3b). However, at saturating *I* (1,000 μmol m^−2^ s^−1^) the process of carboxylation was most influential at preindustrial *C*_a_ (280 µmol/mol), and at present‐day *C*_a_ (400 µmol/mol) the influence of carboxylation was about equal to limiting‐rate selection. This pattern was similar at close to saturating *I* (500 μmol m^−2^ s^−1^), but limiting‐rate selection had a generally higher influence. At low values of *I* (200 μmol m^−2^ s^−1^), the process of electron transport was more influential than carboxylation with the sensitivity of *A* to electron transport increasing as *C*_a_ becomes less limiting. Bimodality was most apparent when limiting‐rate selection was most influential (the alternative modes corresponding with the two alternative representations of limiting‐rate selection).

When all 12 models and all nine environmental scenarios were combined, variation in *A* of more than 5% was caused by only five of the 14 parameters (Figure 3c; Table 4). *V*_cmax_ was the most influential parameter responsible for 35% of the variation in *A*, followed by *K*_c_ with 22%, *θ*_cj_ with 19%, *K*_o_ with 7%, and *a* with 7%.

**TABLE 4:** First order sensitivity to parameters

Variable	*C*_a_	*I*	Model	Mean	Variance	*a*	*a*_jv_	*α*_tpu_	*K*_c_	*K*_o_	*V*_cmax_	*b*_jv_	*b*_rv_	*b*_tv_	*f*	*k_c_*:*k*_o_	*θ*_cj_	*θ*_cjp_	*θ*_j_
*A*	int	int	int	11.49	0.94	0.07	0.00	0.00	0.22	0.07	0.35	0.00	0.01	0.00	0.01	0.01	0.19	0.04	0.02
	280	200	int	7.77	0.44	0.22	0.00	0.00	0.16	0.09	0.05	0.00	0.00	0.00	0.02	0.05	0.29	0.00	0.05
	400	200	int	9.14	0.52	0.39	0.00	0.00	0.07	0.05	0.02	0.00	0.00	0.00	0.03	0.04	0.27	0.01	0.08
	600	200	int	10.30	0.55	0.49	0.00	0.00	0.03	0.03	0.02	0.00	0.01	0.00	0.04	0.02	0.21	0.03	0.10
	280	500	int	9.15	0.69	0.00	0.00	0.00	0.36	0.11	0.37	0.00	0.00	0.00	0.00	0.02	0.13	0.01	0.00
	400	500	int	12.37	1.11	0.00	0.00	0.00	0.28	0.08	0.41	0.00	0.00	0.00	0.00	0.01	0.19	0.03	0.01
	600	500	int	16.07	1.52	0.02	0.00	0.00	0.15	0.05	0.38	0.00	0.03	0.00	0.00	0.01	0.25	0.06	0.03
	280	1,000	int	9.28	0.69	0.00	0.00	0.00	0.38	0.11	0.39	0.00	0.00	0.00	0.00	0.02	0.09	0.01	0.00
	400	1,000	int	12.61	1.12	0.00	0.00	0.00	0.30	0.08	0.44	0.00	0.01	0.00	0.00	0.01	0.14	0.03	0.00
	600	1,000	int	16.67	1.80	0.00	0.00	0.00	0.21	0.05	0.48	0.00	0.01	0.01	0.00	0.01	0.18	0.07	0.00
	int	int	1111	12.47	0.96	0.09	0.00	0.00	0.28	0.09	0.42	0.00	0.01	0.00	0.01	0.02	0.00	0.00	0.08
	int	int	1211	12.47	0.96	0.09	0.00	0.00	0.28	0.09	0.42	0.00	0.01	0.00	0.01	0.02	0.00	0.00	0.08
	int	int	1121	9.82	0.92	0.04	0.00	0.00	0.11	0.04	0.23	0.00	0.01	0.00	0.00	0.01	0.39	0.12	0.04
	int	int	1221	10.47	1.07	0.03	0.00	0.00	0.13	0.04	0.22	0.00	0.01	0.00	0.00	0.01	0.47	0.00	0.04
	int	int	1112	12.28	0.82	0.09	0.01	0.00	0.30	0.10	0.45	0.01	0.03	0.00	0.01	0.02	0.00	0.00	0.00
	int	int	1212	12.28	0.82	0.09	0.01	0.00	0.30	0.10	0.45	0.01	0.03	0.00	0.01	0.02	0.00	0.00	0.00
	int	int	1122	9.65	0.84	0.03	0.00	0.00	0.11	0.04	0.23	0.00	0.01	0.00	0.00	0.01	0.42	0.12	0.00
	int	int	1222	10.27	0.97	0.03	0.00	0.00	0.13	0.04	0.22	0.00	0.01	0.00	0.00	0.01	0.52	0.00	0.00
	int	int	1113	12.82	1.00	0.13	0.00	0.00	0.31	0.09	0.45	0.01	0.00	0.00	0.01	0.02	0.00	0.00	0.00
	int	int	1213	12.82	1.00	0.13	0.00	0.00	0.31	0.09	0.45	0.01	0.00	0.00	0.01	0.02	0.00	0.00	0.00
	int	int	1123	10.80	0.93	0.05	0.00	0.00	0.17	0.05	0.31	0.01	0.00	0.01	0.01	0.01	0.19	0.21	0.00
	int	int	1223	11.67	0.97	0.06	0.00	0.00	0.25	0.07	0.34	0.00	0.00	0.00	0.00	0.01	0.25	0.00	0.00
∆*A*	int	int	int	2.81	0.09	0.09	0.01	0.00	0.09	0.02	0.26	0.00	0.07	0.01	0.01	0.00	0.20	0.11	0.05
	280–400	200	int	1.38	0.07	0.38	0.00	0.00	0.11	0.03	0.17	0.00	0.01	0.00	0.04	0.01	0.02	0.02	0.05
	400–600	200	int	1.16	0.02	0.34	0.00	0.00	0.15	0.09	0.02	0.00	0.01	0.00	0.03	0.07	0.09	0.10	0.07
	280–400	500	int	3.22	0.07	0.01	0.00	0.00	0.07	0.01	0.41	0.00	0.01	0.00	0.00	0.00	0.36	0.10	0.02
	400–600	500	int	3.70	0.21	0.07	0.01	0.00	0.11	0.01	0.10	0.00	0.15	0.00	0.00	0.00	0.13	0.08	0.10
	280–400	1,000	int	3.32	0.07	0.00	0.00	0.00	0.08	0.01	0.47	0.00	0.02	0.01	0.00	0.00	0.30	0.13	0.01
	400–600	1,000	int	4.07	0.12	0.01	0.01	0.00	0.04	0.01	0.42	0.00	0.03	0.02	0.00	0.01	0.27	0.21	0.01
	int	int	1111	3.20	0.10	0.04	0.00	0.00	0.08	0.01	0.29	0.00	0.06	0.00	0.00	0.00	0.00	0.00	0.20
	int	int	1211	3.20	0.10	0.04	0.00	0.00	0.08	0.01	0.29	0.00	0.06	0.00	0.00	0.00	0.00	0.00	0.20
	int	int	1121	2.05	0.09	0.03	0.01	0.00	0.04	0.02	0.08	0.00	0.03	0.02	0.01	0.01	0.33	0.36	0.07
	int	int	1221	2.44	0.10	0.03	0.00	0.00	0.01	0.00	0.09	0.00	0.04	0.00	0.00	0.00	0.65	0.00	0.11
	int	int	1112	3.10	0.11	0.11	0.03	0.00	0.20	0.02	0.29	0.00	0.21	0.00	0.01	0.01	0.00	0.00	0.00
	int	int	1212	3.10	0.11	0.11	0.03	0.00	0.20	0.02	0.29	0.00	0.21	0.00	0.01	0.01	0.00	0.00	0.00
	int	int	1122	1.98	0.07	0.03	0.01	0.00	0.05	0.02	0.08	0.00	0.04	0.02	0.01	0.01	0.36	0.38	0.00
	int	int	1222	2.35	0.08	0.04	0.00	0.00	0.02	0.00	0.09	0.00	0.05	0.00	0.00	0.00	0.74	0.00	0.00
	int	int	1113	3.33	0.10	0.21	0.01	0.01	0.11	0.03	0.55	0.01	0.01	0.01	0.02	0.01	0.01	0.01	0.01
	int	int	1213	3.33	0.10	0.21	0.01	0.01	0.11	0.03	0.55	0.01	0.01	0.01	0.02	0.01	0.01	0.01	0.01
	int	int	1123	2.53	0.11	0.05	0.01	0.01	0.03	0.02	0.15	0.01	0.01	0.05	0.01	0.01	0.12	0.61	0.01
	int	int	1223	3.11	0.07	0.11	0.00	0.00	0.05	0.00	0.35	0.00	0.00	0.00	0.01	0.00	0.45	0.00	0.00

The influence of these parameters somewhat reflects the influence of the processes to which they belong. However, if we were to only consider variability in *A* caused by parameter variation, the influence of carboxylation would be over‐estimated. *V*_cmax_, *K*_o_ and *K*_c_ are all parameters in the process of carboxylation and together they were responsible for 64% of the variation in *A* in the parameter SA (total variance: 0.94). Together *θ*_cj_ and *θ*_cjp_, the parameters of limiting‐rate selection, were responsible for only 23% of variance in the parameter SA. On the other hand, the process SA suggested that carboxylation was responsible for a more modest 22% of the variation in *A* (total variance: 2.59), while limiting‐rate selection was responsible for 57%. The results presented here suggest that variation in *A* between the alternative limiting‐rate selection models (hypotheses) was substantial, and not solely a result of variation in parameters. The difference in variance accounting by the two methods is demonstrated by the difference in variance calculated by the parametric SA (0.94) and the process SA (2.59) despite both algorithms using all possible model combinations and the same parameter ranges (the means of *A* calculated by the algorithms were equivalent: 11.49 and 11.49; Tables 3 and 4).

Sensitivities for individual models (integrated across environmental scenarios) showed that, given equal variation (±10%), *V*_cmax_ and *θ*_cj_ shared similar maximum sensitivities: 48% and 52% respectively (Figure 3e; Table 4). *θ*_cjp_ had a maximum sensitivity of 21% in the model with smoothing, TPU and no *J*_max_ term in electron transport (M1223). Leaf light absorption, *a*, featured in all models of electron transport, and therefore all models, and sensitivity to *a* varied between 9% and 13% or 3%–6% depending on minimum or smoothing limiting‐rate selection respectively. Similarly, *V*_cmax_, *K*_o_ and *K*_c_ were all more influential in the models that used the minimum for limiting‐rate selection. Sensitivities for individual environmental conditions (integrated across models) showed that as expected *a* was influential under low‐light conditions while *V*_cmax_, *K*_o_ and *K*_c_ were influential under high‐light conditions (Figure 3d). Sensitivities for individual model and environmental condition combinations showed that for some cases some of the previously unmentioned parameters were influential (e.g. *θ*_j_, *f*), while others remained with very little influence (e.g. *a*_jv_, *b*_jv_, *b*_tv_, *α*_TPU_).

### 3.2 SA of the assimilation response (Δ*A*) to changes in *C*_a_

**FIGURE 4:**
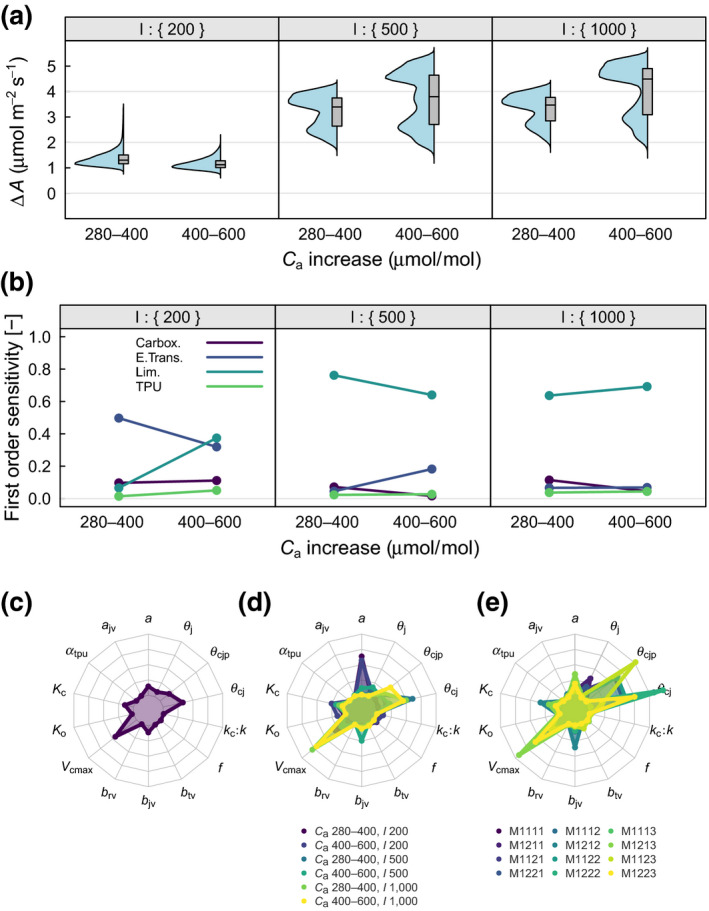
Sensitivity of the carbon assimilation response to an increase in *C*_a_ (Δ*A*, μmol m^−2^ s^−1^) to variability in processes and parameters across *I*. Descriptions the same as for Figure 3

Figure 4a shows the distribution of Δ*A* in response to changes in *C*_a_ (from preindustrial to present‐day, 280–400 µmol/mol, and from present‐day to projected future concentrations, 400–600 µmol/mol) at three levels of *I*. Variation of Δ*A* was greatest at intermediate light levels and going from present to future *C*_a_ (range about 2 to 5 μmol m^−2^ s^−1^). Variation was similar at high light. For both *C*_a_ changes and at both high and intermediate light, the distribution of Δ*A* was highly bimodal, with stronger bimodality going from preset to future *C*_a_. As for *A*, the alternative modes were associated with the alternative representations of limiting‐rate selection.

When all environmental scenarios were combined, limiting‐rate selection was responsible for 65% of the variation in Δ*A* (total variance: 0.50), carboxylation responsible for 5%, electron transport 13% and TPU 3% (Table 3). The strong influence of limiting‐rate selection was borne out across the majority of environmental conditions (Figure 4b). For both *C*_a_ changes and at high and intermediate *I*, sensitivity of Δ*A* to limiting‐rate selection ranged from 64% to 76%, with the higher sensitivities at intermediate *I*. At low *I* electron transport was the most influential process, accounting for 50% of the variation in Δ*A* at the lower *C*_a_ change and 32% at the higher *C*_a_ change. At low *I* sensitivity of Δ*A* to limiting‐rate selection increased from 7% at the lower *C*_a_ to 37% at the higher *C*_a_.

In contrast with the sensitivity of *A*, seven parameters were responsible for over 5% variation in Δ*A* (total variance—0.09) when models and environmental scenarios were combined. Of these seven parameters, four were in common with *A* (*V*_cmax_ with 26%, *K*_c_ with 9%, *θ*_cj_ with 20% and *a* with 9%; Figure 4c; Table 4), *K*_o_ did not feature, and *θ*_cjp_ with 11%, *b*_rv_ with 7% and *θ*_j_ with 5% were also influential.

### 3.3 Consequences of limiting‐rate selection assumptions

**FIGURE 5:**
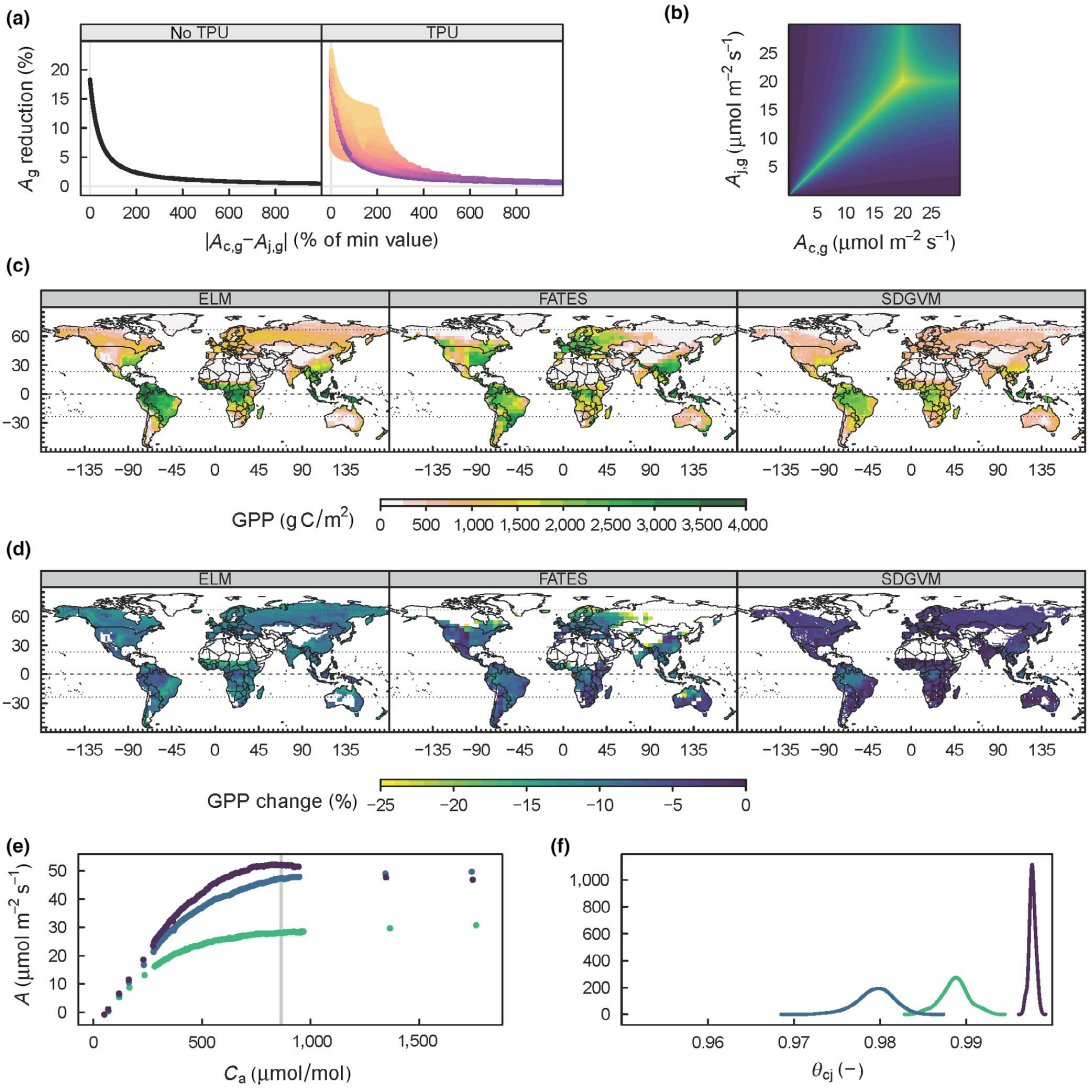
Relative reduction in calculated *A*_g_ of GPP (%) using quadratic smoothing compared to the minimum of the limiting rates. (a) Relative reduction in *A*_g_ (%) against the relative difference in *A*_c,g_ and *A*_j,g_ (% increase relative to the minimum of the two rates) when triose phosphate use is not simulated or simulated, colours represent relative difference in *A*_p,g_ and the minimum of *A*_c,g_ and *A*_j,g_ with orange representing the lowest difference and therefore the largest reduction in *A*_g_. (b) The relative reduction in *A*_g_ (%) as a function of both *A*_c,g_ and *A*_j,g_ when *A*_p,g_ is 20 μmol m^−2^ s^−1^ (see colour scale for d). (c) Global GPP (g C/m^2^) simulated by the three terrestrial biosphere models (TBMs): ELM, FATES and SDGVM. (d) Relative reduction in GPP (%) caused by non‐rectangular hyperbolic smoothing in the three TBMs. (e) High‐resolution *A–C*_a_ curves used to estimate *θ*_cj_. (f) MCMC posterior distributions for *θ*_cj_ estimated from the curves in (e). The vertical grey line in (e) represents the *C*_a_ cutoff for the single high‐resolution curve that showed a drop in *A* at high *C*_a_. Where values of GPP in (c) were less than 250 g C/m^2^, values in (d) were screened to avoid over‐emphasizing high relative changes on small absolute rates of GPP that do not contribute substantially to the global carbon cycle

Given the sensitivity of *A* and Δ*A* to the processes of limiting‐rate selection, we now investigate these models in more detail. Mathematical analysis shows that FvCB sets the upper limit for *A* while CBGB smoothing always reduces *A* below that of the minimum (see Supporting Information). The greatest reduction in *A* caused by smoothing is when all three limiting rates—*A*_c,g_, *A*_j,g_ and *A*_p,g_—are equal, and yields Equation S3 (see Supporting Information). With *θ*_cj_ = 0.95, *θ*_cjp_ = 0.98, Equation S3 shows that the smoothing scalar on *A*_g_ is 0.77, i.e. when *A*_c,g_ *= A*_j,g_ *= A*_p,g_ quadratic smoothing reduces *A*_g_ by 23%. When only *A*_c,g_ and *A*_j,g_ are equal and substantially lower than *A*_p,g_ (so *A*_p,g_ effectively has no influence on smoothing), *A*_g_ is reduced by 18%. Figure 5a shows that *A*_g_ is reduced below the minimum rate across a wide range of *A*_c,g_ and *A*_j,g_ values and that the reduction in *A*_g_ approaches 0 monotonically as the difference between the minimum rate and the larger rate increases.

The results so far are all based on a leaf‐scale SA. To investigate the global‐scale impact of the most influential leaf‐scale processes, a suite of three different TBMs were run with the two alternative versions of limiting‐rate selection, and parameter values for smoothing from CBGB (Figure 5c,d). The three models simulate quite different magnitudes and patterns of global GPP (Figure 5c), and in all three models quadratic smoothing reduced global GPP (5.0–16.4 Pg C/year, or 4.4%–9.7%).

### 3.4 Discriminating among hypotheses for limiting‐rate selection

High‐resolution *A*–*C*_i_ curves were taken on three individuals of *Populus canadensis* (Figure 5e) in order to estimate the *θ*_cj_ parameter, and thus discriminate among the two competing hypotheses for limiting‐rate selection. A value close to 1.0 would indicate FvCB and values close to 0.95 or less would indicate CBGB. Bayesian MCMC estimated *θ*_cj_ for the three curves at 0.98, 0.99 and 0.999 (mean 0.99 ± 0.011 95% CI; Figure 5f). Thus the only data‐driven estimate of *θ*_cj_ made to‐date is not significantly different from 1.0, providing support for the FvCB method of limiting‐rate selection.

## 4 DISCUSSION

A novel, mathematically rigorous, process SA that accounts for both hypothesis (process representation) and parameter variability in common models of photosynthesis has shown that limiting‐rate selection was the most influential process, accounting for 57% of variation in *A* and 65% of variation in Δ*A* in response to a change in CO_2_. For simulating *A*, carboxylation was the next most influential process (which was all due to parameter variability) followed by electron transport. When simulating Δ*A*, electron transport was the next most influential process followed by carboxylation. The process of TPU had almost no influence on simulating either *A* or Δ*A* under the environmental conditions of this analysis. The substantial influence of the non‐mechanistic limiting‐rate selection propagates to global simulation of photosynthesis (reducing mean annual global GPP by 5%–10%) and undermines the mechanistic reasoning for including FvCB and CBGB in TBMs. Analysis of novel, high‐resolution *A*–*C*_i_ curves provides support for the FvCB method of limiting‐rate selection.

### 4.1 The influence of limiting‐rate selection

Finding the smaller roots of the quadratics described by Equations (3a,b) is intended to smooth the abrupt transition between *A*_c,g_, *A*_j,g_ and *A*_p,g_, and has been described as representing co‐limitation between limiting rates. In so doing, smoothing also imposes a reduction of modelled *A* (Figure 5), reducing *A*_g_ by 23% compared with FvCB when all potentially limiting rates are equal and with CBGB parameter values. In models that have chosen to adjust these parameters (e.g. IBIS, JULES; Table 1) the reduction can be even greater. For example, in IBIS the reduction increases to 36% and in JULES to 38% (Equation S3). This scenario, when all potentially limiting rates are equal, results in the greatest smoothing‐related reduction in *A*_g_ and *A*, but it is not an extreme physiological scenario. Potential assimilation rates *A*_c,g_ and *A*_j,g_ are often observed to be close to co‐limiting in saturating light (e.g. Ainsworth et al., 2003; Bernacchi et al., 2005) and the co‐ordination hypothesis assumes that *A*_c,g_ and *A*_j,g_ are equal under mean environmental conditions (Maire et al., 2012). Co‐ordination hypotheses are used in a number of optimization schemes (Smith et al., 2019; Wang et al., 2017), which maintain photosynthesis close to the transition given a changing environment. If combined with quadratic smoothing, these methods would maximize the influence and reduction of *A* caused by smoothing, or may have other untoward consequences (e.g. potentially increasing *V*_cmax_ and hence nitrogen demand).

Until now, the parameter values used in the smoothing function have not been based on data‐driven estimates of their values (to the best of our knowledge). We provide initial data‐driven estimates of *θ*_cj_ (0.99 ± 0.011) using high‐resolution *A–C*_i_ curves, which provide support for the FvCB approach and that smoothing parameters in CBGB and the TBMs which use CBGB are too low. However, the support for the simple FvCB minimum is not definitive, leaving the door open for potential co‐limitation. Additional high‐resolution *A*–*C*_i_ curves collected across a broad range of species and growth conditions would help determine if estimates of *θ*_cj_ could be significantly lower than 1, potentially justifying continued inclusion of quadratic smoothing, albeit that the data presented here suggest it would likely be at a lower level than CBGB and the TBMs that currently use quadratic smoothing.

The global‐scale reduction in GPP caused by quadratic smoothing, demonstrates that leaf‐scale sensitivities propagate through a suite of processes and scales to have global impact in our current generation of TBMs. This propagation of leaf‐scale sensitivities holds true across a spectrum of different model assumptions and representations, including: nutrient cycling (ELM), multi‐layered canopy scaling (FATES, SDGVM) and competition among PFTs (FATES).

Given that models which include quadratic smoothing have subsequently been evaluated against larger‐scale observations (e.g. eddy covariance towers, Bonan et al., 2011), replacing CBGB smoothing with the FvCB minimum could result in a reduction in model skill. Indeed, smoothing, or co‐limitation, among potentially limiting photosynthetic rates reduced GPP and therefore improved simulations of global GPP in CLM4 (Bonan et al., 2011). Bonan et al. (2011) used a number of methods to parameterize *V*_cmax_, all of which were based on *V*_cmax_ values estimated using an FvCB type model. As we have shown, and has been previously demonstrated (Johnson & Thornley, 1984), CBGB smoothing reduces *A* and hence if a CBGB type model had been used to estimate *V*_cmax_, *V*_cmax_ values would have been higher in order to compensate smoothing‐related reductions in *A*. That is to say, estimates of *V*_cmax_ are not independent of the limiting‐rate selection method used in their estimation and that *V*_cmax_ and *θ*_cj_ parameter values applied in TBMs should be consistent with the method used to estimate *V*_cmax_. For example, IBIS, in comparison with other TBMs has very high values for *V*_cmax_ (Rogers, 2014) which may have been required during model calibration to compensate for the use of smoothing in limiting‐rate selection. In models which tie *V*_cmax_ to leaf nitrogen and plant nitrogen demand, the implementation of smoothing would have implications for the coupling of carbon and nitrogen cycles by reducing carbon gained per unit leaf nitrogen.

What process is smoothing intended to represent? CBGB introduce smoothing to represent a more realistic transition in the light response of *A*, and this could represent imperfect coupling among the cycles of electron transport, carboxylation and photorespiration (Farquhar et al., 1980). It is also possible that smoothing is accounting for different limiting states of individual chloroplasts within a leaf (Buckley et al., 2017; Kull & Kruijt, 1998). For canopy scale big‐leaf models, smoothing is also accounting for different limiting states of leaf layers within either the sunlit or shaded canopy fraction. There is also an intermediate scale where smoothing could be accounting for different limiting states of leaves within a canopy leaf layer (also potentially within either the sunlit or shaded fraction). To use a leaf‐scale empirical function as a catch‐all for all of these processes is not satisfying. An 18% non‐mechanistic reduction of *A*_g_ at the light‐limited to light‐saturated transition point is not likely to be an accurate representation of all of these processes. It would be better to use a more mechanistic approach, or a defensible approximation of the mechanistic approach could be developed where computational efficiency is needed.

We suggest removing CBGB smoothing from TBMs on mechanistic grounds and the evidence at hand. However, quadratic smoothing may still be preferred by some TBMs as it provides a continuous derivative to *A* as a function of environment, and is thus preferable for use in numerical solutions to the coupled system of equations that describe *A*. Where quadratic smoothing is preferred, the data suggest a value of 0.99 ± 0.011 for *θ*_cj_ which still would result in a reduction in *A*_g_ at the light‐limited to light‐saturated transition of 9.1%. We recommend at a minimum the higher value of 0.998 used by Buckley et al. (2017) that results in an *A*_g_ reduction of 4.3%.

### 4.2 Influence of other processes

Electron transport, the process most commonly thought of as the key difference among FvCB and CBGB, was not a strongly influential process (10% for *A* and 13% for Δ*A* when integrated across environmental scenarios). The influence of electron transport as a process was not greater than the sum of the influence of its parameters: *a*, *a*_jv_, *b*_jv_, *f* and *θ*_j_ (sum of sensitivities indexes 11% for *A* and 16% for Δ*A*). That is to say, the alternative representations of electron transport did not result in appreciable between‐model variability under the environmental and other conditions of the SA. This result suggests that under the conditions of this SA, a linear electron transport rate or a saturating rate with *J*_max_ simulated as a linear function of *V*_cmax_ had very little effect on simulated assimilation rates. This inference is supported by the small sensitivity indexes of *a*_jv_ and *b*_jv_ (0% and 0% integrated across models and scenarios, for *A*).

Our setup, based on a commonly used relationship in TBMs (Wullschleger, 1993) and a *V*_cmax_ value at the upper end of the range for a tropical PFT (Rogers, 2014), gave a range in the *J*_max_ *:V*_cmax_ ratio at 25°C (JV_25_) of 1.95 to 2.52. This range is fairly high, e.g. Bonan et al. (2011) used a JV ratio of 1.97, and likely contributed to the lower influence of electron transport as a process. A recent analysis showed the JV_25_ ratio to have the global range of approximately 1.0–2.5 (Kumarathunge, Medlyn, Drake, Tjoelker, et al., 2019). Nevertheless, the central values of *a*_jv_ and *b*_jv_ that we used are commonly employed by TBMs and a *V*_cmax_ of 50 μmol m^−2^ s^−1^ is a fairly representative value, so the parameter space of our SA is likely representative of a substantial proportion of parameter space across multiple TBMs.

Electron transport in the FvCB models also uses empirical smoothing between *J*_max_ and an electron transport rate that is linear with *I*. In models that employ non‐rectangular smoothing in electron transport the parameter *θ*_j_ has a sensitivity index of 4%–8% when integrating across environmental scenarios. Smoothing of *J* in response to light has less influence than smoothing for limiting‐rate selection because it only smooths the light response, thus can only influence the light‐limited rate, while CBGB smoothing is applied to every single calculation of *A*. Furthermore the empirical smoothing used to represent the response to *I* is the best representation of that relationship currently available and lacks the mechanistic understanding and representation possible for *A*_c,g_ using Michaelis–Menten kinetic theory (von Caemmerer, 2000).

With the addition of TPU as a limiting rate, smoothing further decreases *A*_g_ compared with using the minimum (Figure 5a). The SA suggested that the inclusion of TPU limitation as a process was not strongly influential (2% for *A* and 3% for Δ*A* when integrated across environmental scenarios). Due to the co‐ordination of photosynthetic apparatus, the rate of TPU export is usually simulated as a proportion of *V*_cmax_ (Equation 9). Collatz et al. (1991) used a value of *b*_tv_ equivalent to 0.167, which is commonly used and is the central value used in this SA. Lombardozzi et al. (2018) pointed out that this value of *b*_jv_ may be too high based on Wullschleger (1993) and demonstrated a 9 Pg C (about 9%) smaller increase in global terrestrial ecosystem C between 1850 and 2100 in CLM4.5 under RCP8.5 when a value of 0.083 was used for *b*_jv_. However, 0.083 is >1 standard error lower than the Wullschleger (1993) mean and lower than the 95% CI at 25°C from a recent synthesis (Kumarathunge, Medlyn, Drake, Rogers, & Tjoelker, 2019). Ellsworth et al. (2015) showed that TPU can be limiting under high‐light and high‐CO_2_ conditions, concluding with a general recommendation that modellers interested in simulating *A* should consider TPU limitation (as formulated by Equation 7). However, their results demonstrate that TPU limitation is primarily influential under conditions of low O_2_ (2%) or saturating *C*_i_ (>100 Pa). Given that these conditions are rather extreme, the low ratio of TPU to *V*_cmax_ chosen by Lombardozzi et al. (2018), and the results of our SA, we suggest that calculating TPU at the photosynthetic core of TBMs is probably an unnecessary computational cost.

Despite the relative lack of influence of the process of carboxylation, *V*_cmax_ was still the most influential parameter (i.e. accounted for more of the within‐model variance than any other parameter) when models and environment were combined. This discrepancy highlights the importance of considering the variability in model process representation when conducting model SA, as illustrated by the different variances calculated by the two SA types (for *A*, parameter SA variance = 0.94, while process SA variance = 2.59, despite equal means).

## 5 CONCLUSIONS

At the heart of TBMs lies the surprising dominance of the non‐mechanistic, limiting‐rate selection process. While empirical smoothing among limiting photosynthetic rates may account for a number of mechanistic processes at various scales, it is unsatisfying that empirical functions have such influence in a model that is intended to be highly mechanistic. Indeed the FvCB model is at the core of many TBMs specifically because of its mechanistic simulation of the primary response of the terrestrial biosphere to rising CO_2_ concentration, a principal driver of global change. In this SA, limiting‐rate selection accounts for 65% of the variance in the CO_2_ response of *A*. That this empirically driven variation lies within what is assumed to be a highly mechanistic process representation at the core of TBMs, it is perhaps not surprising that there is such a vast range of disagreement in Earth System model projections of the future terrestrial carbon sink. While FvCB limiting‐rate selection represents selection of *A* at its upper bound, we suggest that this is a more defensible assumption than a highly influential non‐mechanistic function with an essentially arbitrary choice of parameter values that are not supported by data.

To increase confidence in our understanding and future projections of the carbon cycle, and thus climate, we need to understand how the process representations and parameters used by TBMs drive variation in TBM simulations (Medlyn et al., 2015). Previous methods to evaluate process representations have relied on model inter‐comparison projects (MIPs) either with multiple models (e.g. Anav et al., 2015; Arora et al., 2019; Walker, Hanson, et al., 2014) or comparison of alternative representations of submodels or processes within a single higher‐level system model (e.g. Burrows et al., 2020). Both of these methods sample only an extremely small fraction of possible model combinations (Abramowitz & Bishop, 2014; Fisher & Koven, 2020). By allowing a fully factorial combination of models, MAAT and the process‐level SA (that includes hypothesis and parameter variability) in this study represents a new frontier for model analysis and development. We investigated a small but influential component of TBMs, finding a surprising leaf‐scale sensitivity that has global‐scale implications. Yet the analysis presented here is just the beginning of what is possible. The quantitative multi‐hypothesis tools provided by MAAT, and by other multi‐hypothesis modelling groups, will help to provide rigorous advances in process‐level understanding of the dynamics of complex ecosystems.

## Supporting information

Supplementary Material

## Data Availability

The Multi‐Assumption Architecture and Testbed (MAAT) code is freely available (https://github.com/walkeranthonyp/MAAT). MAAT and TBM data, and postprocessing scripts are archived on the ESS‐DIVE archive (https://data.ess‐dive.lbl.gov/view/doi:10.15485/1682250, https://data.ess‐dive.lbl.gov/view/doi:10.15485/1682253). The raw *A–C*_i_ data are freely available on the NGEE‐Tropics project data archive (http://doi.org/10.15486/ngt/1674983).
