# Supplementary material for: Multi‐hypothesis comparison of Farquhar and Collatz photosynthesis models reveals the unexpected influence of empirical assumptions at leaf and global scales
Source: Glob Chang Biol. 2020 Oct 31;27(4):804–22. doi: 10.1111/gcb.15366 (PMC7894311; doi:10.1111/gcb.15366)
Supplement: Supplementary file 1 — Supplementary Material [file GCB-27-804-s001.docx]

Supporting Information for:

Multi-hypothesis comparison of Farquhar and Collatz photosynthesis models reveals the unexpected influence of empirical assumptions at leaf and global scales

Running title: Multi-hypothesis analysis of photosynthesis

Anthony P. Walker^1^, Abbey L. Johnson^1^, Alistair Rogers^2^, Jeremiah Anderson^2^, Robert A. Bridges^3^, Rosie A. Fisher^4,5^, Dan Lu^6^, Daniel M. Ricciuto^1^, Shawn P. Serbin^2^, Ming Ye^7^

# Notes S1. Additional methods

## Terrestrial Biosphere Model simulations

E3SM (release: v1.1.0, https://github.com/E3SM-Project/E3SM) (Burrows et al., in review; Yang et al., 2019) was used that incorporates biogeochemistry improvements to ELM, including phosphorus cycling, and carbon and nutrient storage pools. Photosynthesis is represented using that from the Community Land Model version 4.5 (CLM4.5) (Bonan et al., 2011; Oleson et al., 2013). Unlike previous versions of ELM and CLM4.5, nutrient limitation no longer directly down-regulates GPP. Instead, carbon that cannot be allocated to plant structural components due to nutrient limitation is allocated to a non-structural carbohydrate pool, which respires to the atmosphere with a given turnover time (Metcalfe et al., 2017). Stand-alone (offline) ELM simulations were performed at 1.9^o^ x 2.5^o^ spatial resolution. Carbon and nitrogen pools were equilibrated under pre-industrial conditions using the standard 250 years of accelerated spin-up (Thornton & Rosenbloom, 2005), followed by 600 years of further spin-up to equilibrate the phosphorus pools (Yang et al., 2019). Transient input data were then applied from 1850-2010 including historically varying atmospheric CO_2_, the Global Soil Wetness Project 3 (GSWP3) meteorological forcing (van den Hurk et al., 2016), nitrogen deposition (Lamarque et al., 2005), and land use change (Lawrence et al., 2018). The meteorological years 1901-1920 were cycled throughout spin-up, and the transient simulation until 1921. The mean of the last 20 years of the simulations (1991-2010) were analysed.

FATES (tag: sci.1.30.0_api.8.0.0, https://github.com/NGEET/fates) (Fisher et al., 2015; Koven et al., 2019), currently a carbon and water cycle model, was run coupled to CLM (version 5, https://github.com/ESCOMP/CTSM) (Lawrence et al., 2019). Stand-alone CLM(FATES) simulations were run at 4^o^ x 5^o^ spatial resolution to give an illustration of the geographical impact of the photosynthetic smoothing. A constant CO_2_ concentration of 384 µmol mol^-1^ was used and simulations were driven using the GSWP3 forcing (van den Hurk et al., 2016). A low-complexity global PFT configuration was used to test the impact of the smoothing changes on global photosynthesis, without needing to attribute differences between regions to arbitrary differences in plant functional type (PFT) parameterization, a simplified PFT configuration was used. Three universal plant functional types (PFTs; all C3, broadleaf, evergreen trees) varied along a growth-risk axis: fast, medium, and slow, and were present everywhere. The slow growing PFT was less susceptible to disturbance hazards (drought and fire) whereas the fast growing PFT was a better competitor for light and water. The drought and fire tolerant PFT was able to grow in all vegetated areas of the globe, while the fast growing PFT provided more realistic productivity and leaf area index (LAI) for forest areas. CLM(FATES) was spun up from bare ground initialized with all three plant functional types until the model was in LAI equilibrium, whereafter there was no trend in photosynthesis. The average from years 40-50 post LAI equilibrium was analyzed.

SDGVM (tag: Walkeretal2020_GCB, https://bitbucket.org/walkeranthonyp/sdgvm) (Walker et al., 2017; Woodward and Lomas, 2004) is a daily timestep, carbon and water cycle model. SDGVM simulations were performed at 1.0^o^ x 1.0^o^ spatial resolution. Carbon pools were equilibrated under pre-industrial conditions by running 500 years of standard spin-up, recycling 1901-1920 of the meteorological dataset at CO_2_ of 280 µmol mol^-1^ . Transient input data were then applied from 1860-2017 including historically varying atmospheric CO_2_ (Dlugokenky & Tans, 2017), the CRU-JRA meteorological forcing (Le Quéré, et al., 2018), and Hyde land use change dataset (Klein Goldewijk et al., 2017). The means of the simulation years 1991-2010 were analysed.

## Analysis of double quadratic smoothing

To investigate quadratic smoothing employed by CBGB, we analyze the mathematical functions and derive an analytical solution for the maximum reduction in *A_g_* caused by smoothing. When using quadratic smoothing, *A_g_* is always less than the minimum of *A_c,g_*, *A_j,g_*, and *A_p,g_*. The general form of Eqs 3a,b (and 6a) is:

$y\text{ = }\theta x^{2}-\left( a_{1}\text{+}a_{2} \right)x\text{ + }a_{1}a_{2}$. (Eq. S1)

Rewriting Eq 11 gives:

$y\text{ = }\left( x-a_{1} \right)\left( x-a_{2} \right)-\left( 1-\theta\right)x^{2}$. (Eq. S1a)

With a smoothing parameter *0 < θ < 1* and assuming that *a_1_* ≤ *a_2_*  (without loss of generality)*,* *y* is positive for large negative values of *x* and *y(a_1_) < 0*, implying by the Intermediate Value Theorem that the smaller root of *y* is less than *a_1_.* Consequently, *A_cj,g_* is strictly less than the minimum of *A_c,g_* and *A_j,g_*. Similarly, the analogous argument on Eq 3b proves that *A_g_* is strictly less than the minimum of *A_cj,g_* and *A_p,g_.*

The reduction in *A_g_* below the minimum rate is greatest when *A_c,g_*, *A_j,g_*, and *A_p,g_* are equal (mathematical proof not shown). Combining Eq 3a and 3b and solving for *A_g_* when *A_c,g_*, *A_j,g_*, and *A_p,g_* take the same value, *a*. By rearranging the quadratic solution for Eq 11 when *a_1_* = *a_2_* = *a*, it can be shown that the smaller root is *x = ag*(*θ)*, where:

$g\left( \theta\right)\text{ = }\left( 1-\sqrt{\left( 1-\theta\right)} \right)/\theta$. (Eq. S2)

Thus the smaller root of Eq 3a, *A_cj,g_ = ag(θ_cj_)*, and substitution into Eq 3b and solving for the lesser root gives the function:

$A=a\frac{\left( 1\text{+}g\left( \theta_{cj} \right) \right)\sqrt{1-4\theta_{cjp}g\left( \theta_{cj} \right)/{\left( g\left( \theta_{cj} \right)\text{+}1 \right)^{2}}}}{2\theta_{cjp}}$. (Eq. S3)

## **References**

1. Bonan, G. B., Lawrence, P. J., Oleson, K. W., Levis, S., Jung, M., Reichstein, M., et al. (2011). Improving canopy processes in the Community Land Model version 4 (CLM4) using global flux fields empirically inferred from FLUXNET data. *Journal of Geophysical Research: Biogeosciences*, *116*(G2), G02014. <https://doi.org/10.1029/2010JG001593>
2. Burrows, S. M., & et al.,. (n.d.). The DOE E3SM coupled model v1.1 biogeochemistry configuration: overview and evaluation of coupled carbon-climate experiments.
3. Dlugokenky, E., & Tans, P. (n.d.). Global Monitoring Laboratory - Global Greenhouse Gas Reference Network. Retrieved April 15, 2020, from https://www.esrl.noaa.gov/gmd/ccgg/trends/gl_data.html
4. Fisher, R. A., Muszala, S., Verteinstein, M., Lawrence, P., Xu, C., McDowell, N. G., et al. (2015). Taking off the training wheels: the properties of a dynamic vegetation model without climate envelopes, CLM4.5(ED). *Geosci. Model Dev.*, *8*(11), 3593–3619. <https://doi.org/10.5194/gmd-8-3593-2015>
5. van den Hurk, B., Kim, H., Krinner, G., Seneviratne, S. I., Derksen, C., Oki, T., et al. (2016). LS3MIP (v1.0) contribution to CMIP6: the Land Surface, Snow and Soil moisture Model Intercomparison Project – aims, setup and expected outcome. *Geoscientific Model Development*, *9*(8), 2809–2832. <https://doi.org/10.5194/gmd-9-2809-2016>
6. Klein Goldewijk, K., Beusen, A., Doelman, J., & Stehfest, E. (2017). Anthropogenic land use estimates for the Holocene – HYDE 3.2. *Earth System Science Data*, *9*(2), 927–953. <https://doi.org/10.5194/essd-9-927-2017>
7. Koven, C. D., Knox, R. G., Fisher, R. A., Chambers, J., Christoffersen, B. O., Davies, S. J., et al. (2019). Benchmarking and Parameter Sensitivity of Physiological and Vegetation Dynamics using the Functionally Assembled Terrestrial Ecosystem Simulator (FATES) at Barro Colorado Island, Panama. *Biogeosciences Discussions*, 1–46. <https://doi.org/10.5194/bg-2019-409>
8. Lamarque, J.-F., Kiehl, J. T., Brasseur, G. P., Butler, T., Cameron‐Smith, P., Collins, W. D., et al. (2005). Assessing future nitrogen deposition and carbon cycle feedback using a multimodel approach: Analysis of nitrogen deposition. *Journal of Geophysical Research: Atmospheres*, *110*(D19). <https://doi.org/10.1029/2005JD005825>
9. Lawrence, P. J., Lawrence, D. M., & Hurtt, G. C. (2018). Attributing the Carbon Cycle Impacts of CMIP5 Historical and Future Land Use and Land Cover Change in the Community Earth System Model (CESM1). *Journal of Geophysical Research: Biogeosciences*, *123*(5), 1732–1755. <https://doi.org/10.1029/2017JG004348>
10. Le Quéré, C., Andrew, R. M., Friedlingstein, P., Sitch, S., Hauck, J., Pongratz, J., et al. (2018). Global Carbon Budget 2018. *Earth System Science Data*, *10*(4), 2141–2194. <https://doi.org/10.5194/essd-10-2141-2018>
11. Metcalfe, D. B., Ricciuto, D., Palmroth, S., Campbell, C., Hurry, V., Mao, J., et al. (2017). Informing climate models with rapid chamber measurements of forest carbon uptake. *Global Change Biology*, *23*(5), 2130–2139. <https://doi.org/10.1111/gcb.13451>
12. Oleson, K. W., Lawrence, D. M., Bonan, G. B., Levis, S., Swenson, S. C., Thornton, P. E., et al. (2013). *Technical Description of version 4.5 of the Community Land Model (CLM)* (NCAR Technical Note No. TN-503+STR). Boulder, CO, USA: National Centre for Atmospheric Research.
13. Thornton, P. E., & Rosenbloom, N. A. (2005). Ecosystem model spin-up: Estimating steady state conditions in a coupled terrestrial carbon and nitrogen cycle model. *Ecological Modelling*, *189*(1–2), 25–48. <https://doi.org/10.1016/j.ecolmodel.2005.04.008>
14. Walker, A. P., Quaife, T., van Bodegom, P. M., De Kauwe, M. G., Keenan, T. F., Joiner, J., et al. (2017). The impact of alternative trait-scaling hypotheses for the maximum photosynthetic carboxylation rate (Vcmax) on global gross primary production. *New Phytologist*, *215*(4), 1370–1386. <https://doi.org/10.1111/nph.14623>
15. Woodward, F. I., & Lomas, M. R. (2004). Vegetation dynamics – simulating responses to climatic change. *Biological Reviews*, *79*(3), 643–670. <https://doi.org/10.1017/S1464793103006419>
16. Yang, X., Ricciuto, D. M., Thornton, P. E., Shi, X., Xu, M., Hoffman, F., & Norby, R. J. (2019). The Effects of Phosphorus Cycle Dynamics on Carbon Sources and Sinks in the Amazon Region: A Modeling Study Using ELM v1. *Journal of Geophysical Research: Biogeosciences*, *124*(12), 3686–3698. <https://doi.org/10.1029/2019JG005082>
